# Estrogen receptor β2 induces proliferation and invasiveness of triple negative breast cancer cells: association with regulation of PHD3 and HIF-1α

**DOI:** 10.18632/oncotarget.20635

**Published:** 2017-09-04

**Authors:** Lucia Bialesova, Li Xu, Jan-Åke Gustafsson, Lars-Arne Haldosen, Chunyan Zhao, Karin Dahlman-Wright

**Affiliations:** ^1^ Department of Biosciences and Nutrition, Novum, Karolinska Institutet, Huddinge S-141 83, Sweden; ^2^ Center for Nuclear Receptors and Cell Signaling, Department of Biology and Biochemistry, University of Houston, Houston, TX 77204-5056, USA

**Keywords:** ERβ2, ERβ isoform, breast cancer, gene expression profile, PHD3

## Abstract

The two estrogen receptor (ER) subtypes, ERα and ERβ, belong to the nuclear receptor superfamily. The human ERβ variant ERβ2 is proposed to be expressed at higher levels than ERβ1 in many breast tumors and it has been suggested that ERβ2, in contrast to ERβ1, is associated with aggressive phenotypes of various cancers. However, the role of endogenous ERβ2 in breast cancer cells remains elusive. In this study, we identified that triple negative breast cancer (TNBC) cell lines express endogenous ERβ2, but not ERα or ERβ1. This allows novel studies of endogenous ERβ2 functions independent of ERα and ERβ1. We show that overexpression of ERβ2 in TNBC cells increased whereas knockdown of endogenous ERβ2 decreased cell proliferation and cell invasion. To elucidate the molecular mechanism responsible for these cellular phenotypes, we assayed ERβ2 dependent global gene expression profiles. We show that ERβ2 decreases prolyl hydroxylase 3 (PHD3) gene expression and further show that this is associated with increased hypoxia inducible factor 1α (HIF-1α) protein levels, thus providing a possible mechanism for the invasive phenotype. These results are further supported by analysing the expression of ERβ2 and PHD3 in breast tumor samples where a negative correlation between ERβ2 and PHD3 expression was observed. Together, we demonstrate that ERβ2 has an important role in enhancing cell proliferation and invasion, beyond modulation of ERβ and ERβ1 signalling which might contribute to the invasive characteristics of TNBC. The invasive phenotype could potentially be mediated through transcriptional repression of PHD3 and increased HIF-1α protein levels.

## INTRODUCTION

Breast cancer is the most frequently diagnosed cancer among women in industrialized countries [1]. According to immunohistochemical staining of estrogen receptor α (ERα), progesterone receptor (PR) and human epidermal growth factor receptor 2 (HER2), breast cancer is categorized into the following groups; Luminal, subtype A: ERα+/PR+/HER2-; luminal, subtype B: ERα+/PR+/HER2+; HER2 overexpression: ERα-/PR-/HER2+ and triple-negative breast cancer (TNBC): ERα-/PR-/HER2- [2]. TNBC accounts for approximately 15 to 20% of all breast cancers and is often more aggressive with higher rates of recurrence and more frequent distant metastasis than other types of breast cancer [3]. Due to a lack of targeted therapy options available to subgroups that express ERα or with HER2 amplification, cytotoxic chemotherapy is the systemic therapy currently available for TNBC patients [4, 5]. Thus, there is a clear unmet medical need to identify new therapeutic targets for TNBC and understanding the mechanism of their action.

ERs belong to the nuclear receptor protein superfamily of ligand-activated transcription factors [6, 7]. For a long time, only one ER, ERα, was thought to exist [8]. However, in 1996, we reported a second ER, ERβ [9]. Like many other genes, ERβ is expressed as different isoforms, the functions of which need to be studied in order to understand the physiological functions of ERβ. The ERβ protein that shares the domain structure of other nuclear receptors is referred to as the full length wild type ERβ or ERβ1. The most studied human variant of ERβ, ERβ2, is the result of alternative splicing, the last 61 amino acids of ERβ1 being replaced by 26 unique amino acids from an alternative last exon. ERβ2 lacks an intact ligand binding domain and activation function 2 (AF-2). Since ERβ2 has an intact DNA-binding domain and an intact N-terminal domain, including the AF-1 region, it could be involved in gene regulation.

ERα has been shown to generally promote growth of ERα-positive breast cancer, which forms the basis for the use of ER antagonists as first line therapy in this breast cancer subgroup. ERβ1 has been suggested to display anti-proliferative properties, including in breast cancer [10, 11]. The prognostic impact of ERβ2 expression in breast cancer remains controversial. Several studies suggest that high ERβ2 expression is associated with poor outcome in breast cancer [12]. In contrast, some studies have reported that ERβ2 was associated with good clinical outcome or no prognostic value in breast cancer [13–16]. In a recent publication, Andersson et al show that most used ERβ antibodies are not sufficiently specific, providing a potential explanation for the apparent discrepancy of certain published studies [17]. Studies of the molecular mechanism of ERβ2 action have been limited by the lack of cellular models that express ERβ2, alone or in the presence of ERα and/or ERβ1. We have previously demonstrated, using a derivate of the ERα-positive breast cancer cell line MCF-7 engineered to express ERβ2, that ERβ2 heterodimerizes with ERα and inhibits ligand induced ERα transcriptional activity by inducing proteasome-dependent degradation of ERα [18]. However, the possible role of endogenous ERβ2 in breast cancer cells remains to be determined.

HIF-1α is a major determinant of invasion and metastasis in a wide variety of tumor types including breast cancer [19]. Its expression is regulated through modifications at the posttranslational level. In the presence of oxygen, three prolyl hydroxylase (PHD) enzymes, PHD1, PHD2 and PHD3, can hydroxylate two HIF-1α proline residues (p402 and p564) in the oxygen-dependent degradation domain. Hydroxylated HIF-1α is recognized by the tumour suppressor von Hippel-Lindau protein, which targets HIF-1α for degradation [20].

In this study, we identify TNBC breast cancer cell lines that express endogenous ERβ2 but not ERα or ERβ1. We then explore BT549 and MDA-MB-231 cell lines to approach the function of ERβ2 independently of ERα and ERβ1. Our results show that ERβ2, when expressed alone, promotes cell proliferation and invasion. We also investigate ERβ2 dependent global effects on gene expression and suggest a mechanism by which ERβ2 contributes to an invasive phenotype. Whether our results can be translated into an understanding of the molecular mechanism for the invasive nature of TNBC and potentially the identification of targeted therapies remains to be determined.

## RESULTS

### Triple-negative breast cancer (TNBC) cell lines express high level of ERβ2

**Figure 1:**
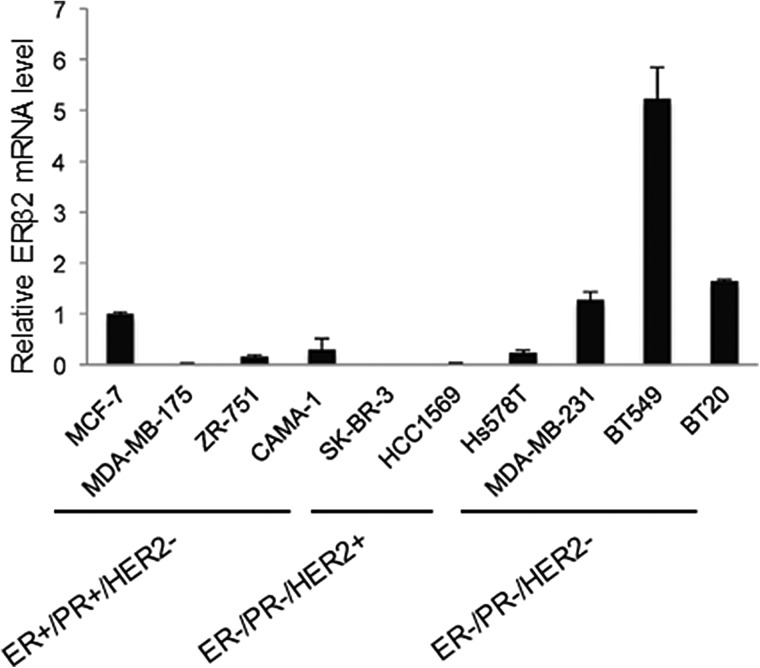
The TNBC cell lines BT549 and MDA-MB-231 express endogenous ERβ2 Analysis of ERβ2 mRNA levels in ER+/PR+/HER2- breast cancer cell lines (MCF-7, MDA-MB-175, ZR-751 and CAMA-1), ER-/PR-/HER2+ breast cancer cell lines (SK-BR-3 and HCC1569) and ER-/PR-/HER2- TNBC cell lines (Hs578T, MDA-MB-231, BT549 and BT20) by qPCR. mRNA levels are normalized to GAPDH, and mRNA levels are presented as means ± SD, relative to the expression level in MCF-7 cells.

We screened the expression of ERβ2 in a panel of ten breast cancer cell lines, including four ER-positive luminal (*MCF-7, MDA-MB-175, ZR-751, CAMA-1*), two HER2-positive (*SK-BR-3 and HCC1569*) and four TNBC (*Hs578T, MDA-MB-231, BT549 and BT20*) cell lines. The highest level of endogenous ERβ2 expression was observed in the BT549, MDA-MB-231 and BT20 TNBC cell lines (Figure 1). The mRNA expression levels of ERβ1 and ERα in these cell lines were undetectable (data not shown). BT549 and MDA-MB-231 cells were used for further functional studies due to the possibility to achieve high transfection efficiency in these two cell lines.

### Depletion of ERβ2 inhibits cellular proliferation and invasion *in vitro*

**Figure 2:**
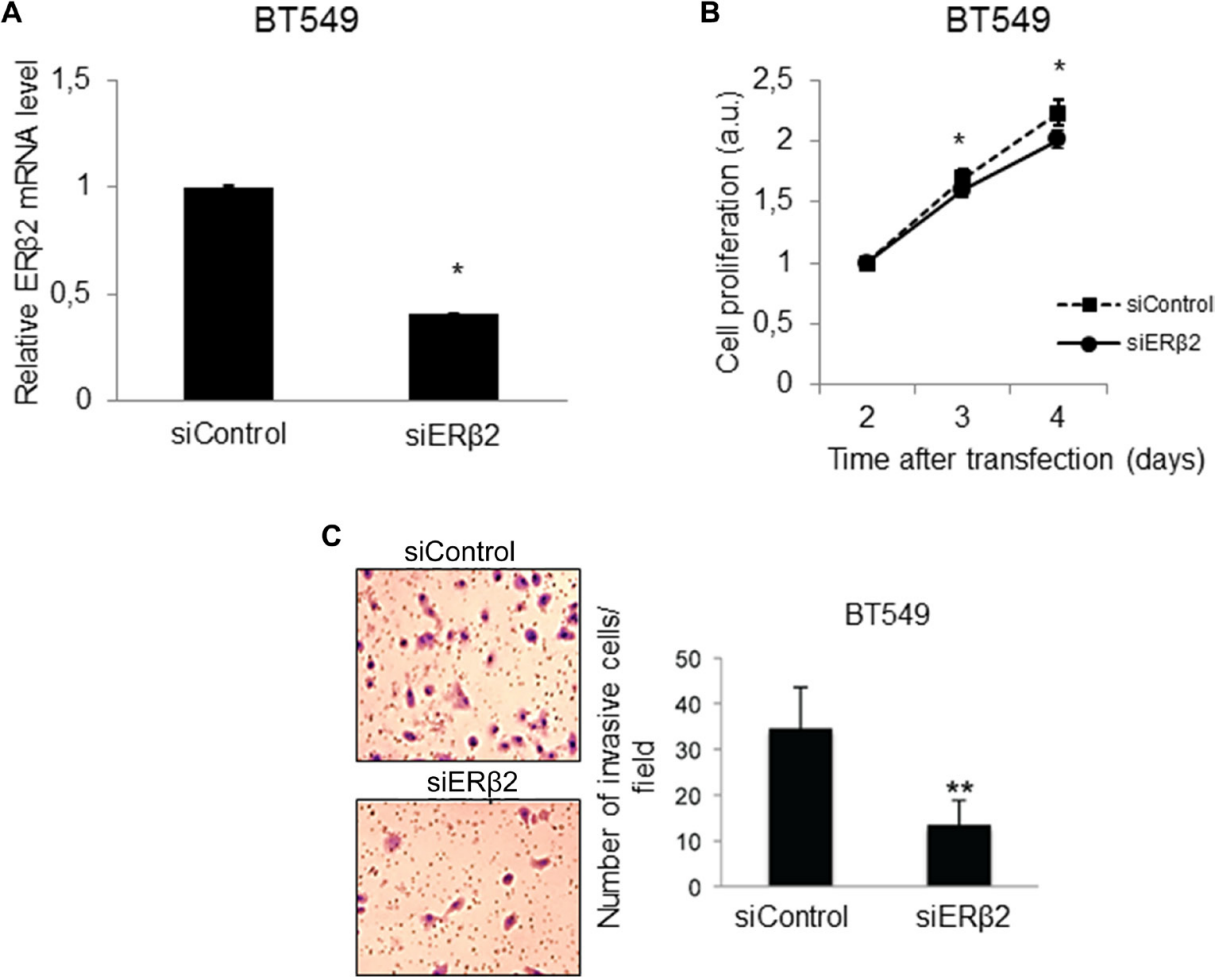
Depletion of ERβ2 inhibits cellular proliferation and invasion (**A**) ERβ2 siRNA down-regulates ERβ2 mRNA in BT549 cells. ERβ2 mRNA level was determined by qPCR after transfection with control siRNA or ERβ2 siRNA. Data are normalized to 36B4 and shown as relative fold change compared to control siRNA ± SD. *P < 0.05. (**B**) ERβ2 depletion reduces proliferation of the BT549 cell line. BT549 cells were transfected with control siRNA or ERβ2 siRNA. WST-1 assays as a measure of cellular proliferation were carried out at the indicated time points after siRNA transfection. Ratio of absorbance to day 1 is calculated. Data are shown as means ± SD. *P < 0.05. The experiment was repeated three times. One representative experiment is shown. (**C**) ERβ2 depletion reduces invasion of BT549 cell line. BT549 cells were transfected with control siRNA or ERβ2 siRNA, and cell invasion was evaluated by the BD Biocoat growth factor reduced Matrigel invasion chamber assay. Data represent means ± SD. **P < 0.01. Experiment was repeated twice. One representative experiment is shown. A, B, C, *p* values were calculated by *t*-test.

We used siRNA to silence ERβ2 expression in BT549 and MDA-MB-231 cells. Successful knockdown of ERβ2 was confirmed at the mRNA level (Figures 2A and S1A). Interestingly, ERβ2 knockdown decreased proliferation of both BT549 (Figure 2B) and MDA-MB-231 cells (Supplementary Figure 1B). Moreover, in invasion assays, ERβ2 knockdown significantly reduced the number of invading cells, from 34.6 ± 9.0 cells per field after control siRNA transfection to 13.4 ± 5.5 cells per field after ERβ2 siRNA transfection of BT549 cells (Figure 2C) (*p* < 0.01). These results were further validated in the MDA-MB-231 cell line where the number of invading cells per field was 12.7 ± 4.5 in the control siRNA transfected cells and 4.1 ± 3.1 in the ERβ2 siRNA transfected cells (Supplementary Figure 1C) (*p* < 0.001). The inhibition of invasion was also confirmed with a second set of siRNA targeting ERβ2 (data not shown), supporting that the observed effects are not related to off-target effects.

### ERβ2 overexpression confers a more proliferative and invasive phenotype *in vitro*

**Figure 3:**
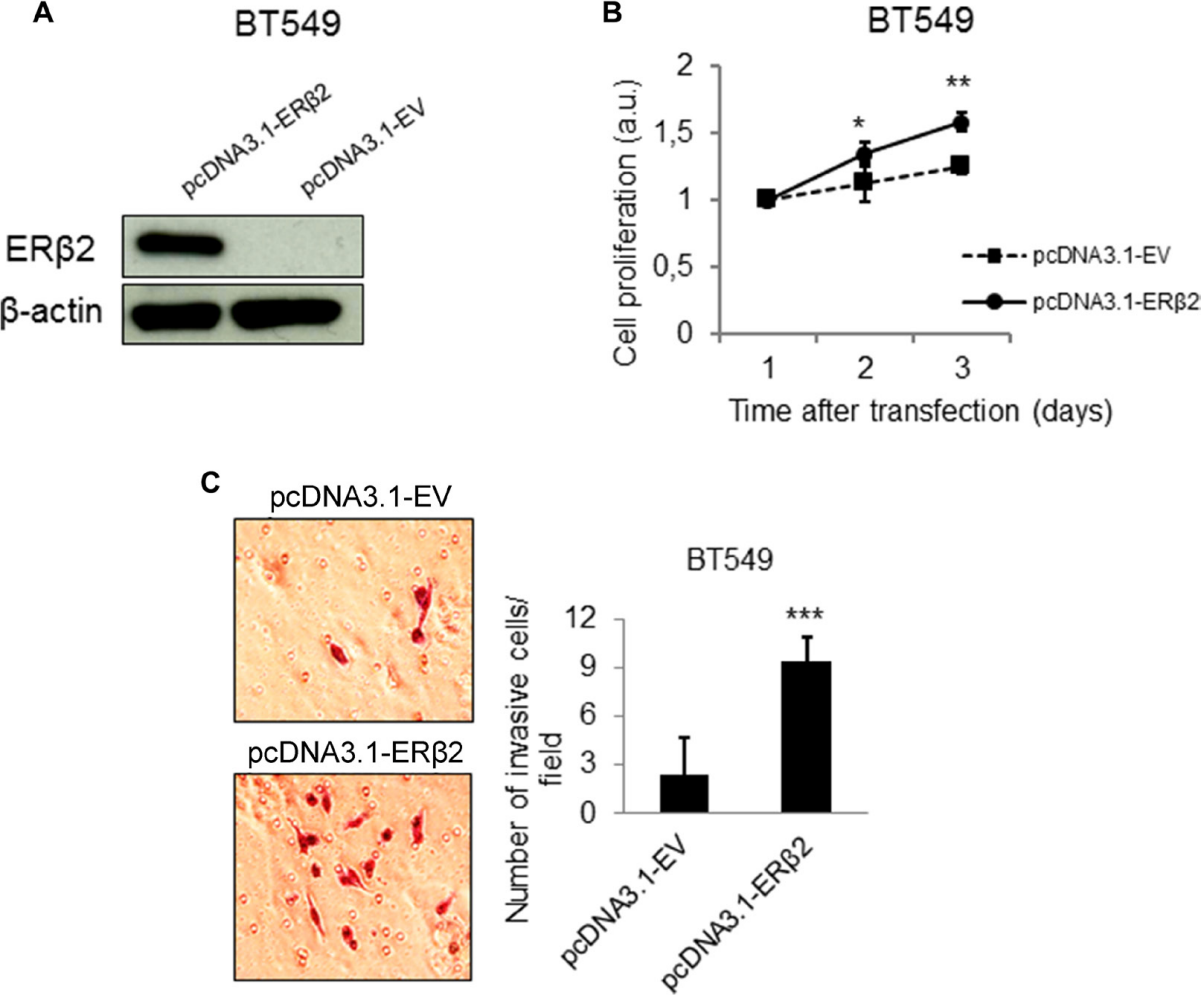
ERβ2 overexpression confers a more proliferative and invasive phenotype *in vitro* (**A**) Western blot analysis showing increased protein level of ERβ2 after transient overexpression of ERβ2 protein. ERβ2 was detected by the PPZ0506 antibody. β-actin was used as a loading control. (**B**) ERβ2 overexpression promotes cell proliferation in the BT549 cells. WST-1 assays of cell proliferation were carried out at the indicated time points after transfection of ERβ2 or empty vector (EV). Ratio of absorbance to day 1 is calculated. Data are shown as means of relative absorbance ± SD. *P < 0.05, **P < 0.01. Experiments were repeated three times. One representative experiment is shown. (**C**) ERβ2 overexpression promotes cell invasion in the BT549 cell line. BT549 cells were transfected with ERβ2 or EV, and cell invasion was evaluated by the BD Biocoat growth factor reduced Matrigel invasion chamber assay. Data represent means ± SD. ***P < 0.001. Experiment was repeated twice. One representative experiment is shown. B,C, *p* values were calculated by *t*-test.

To further support the effects of ERβ2 on cellular proliferation and invasion, we investigated these phenotypes after ERβ2 overexpression. Successful overexpression of ERβ2 was confirmed by Western blot analysis (Figures 3A and S2A). Importantly, ERβ2 overexpression promoted cell proliferation in both investigated TNBC cell lines (Figures 3B and S2B). In addition, cells overexpressing ERβ2 acquired a more invasive phenotype with 9.4 ± 2.3 cells migrating through the chamber for ERβ2 overexpression cells compared to 2.4 ± 1.4 for the control cells for the BT549 cell line (Figure 3C) (*p* < 0.001). Similarly, overexpression of ERβ2 in MDA-MB-231 cells significantly increased cell invasion with 11.3 ± 5.9 invading cells per field for ERβ2 overexpression cells compared to 2.5 ± 1.8 invading cells for the control cells (Supplementary Figure 2C) (*p* < 0.001). These results further support the link between ERβ2 levels and cellular proliferation and invasion.

### ERβ2 effects on global gene expression profiles

To approach the molecular mechanism responsible for the effects of ERβ2 on cellular phenotypes, we determined changes in gene expression profiles for BT549 cells upon ERβ2 knockdown. We identified 662 genes, applying a false discovery rate of less or equal to 0.1, as upregulated (fold change equal or greater than 1.5, *p* < 0.05) while the expression of 263 genes was repressed (fold change equal or less than 1.5, *p* < 0.05) upon ERβ2 knockdown. Network analysis revealed the top three ranked networks regulated by inhibiting endogenous ERβ2 in BT549 cells as cell morphology, DNA replication and repair, and cell death and survival (Table 1). Molecular and cellular functional classification analysis shows how alterations of gene expression were predicted to disrupt various molecular and cellular functions. The top 5 highlighted molecular and cellular functions after ERβ2 knockdown were cell cycle, cell death and survival, morphology, development and organization (Table 2).

**Table 1:** Changed networks after knockdown of ERβ2 in BT549 cells

Top Networks		
*ID*	Associated Network Functions	Score
1	Cell Morphology, DNA Replication, Recombination and Repair, Developmental Disorder	32
2	Cancer, Reproductive System Disease, Cell Dearth and Survival	24
3	Cell Morphology, Dermatological Diseases and Conditions, Developmental Disorder	24

**Table 2:** Changed molecular functions after knockdown of ERβ2 in BT549 cells

Name	*p*-value	#Molecules
Cell Cycle	1.94E-03–4.01E-02	5
Cell Death and Survival	1.94E-03–4.46E-02	5
Cell Morphology	1.94E-03–4.57E-02	8
Cellular Development and Organization	1.94E-03–3.63-02	7
Cellular Development	1.94E-03–3.79-02	4

### Validation of global gene expression profiling data by qPCR

**Figure 4:**
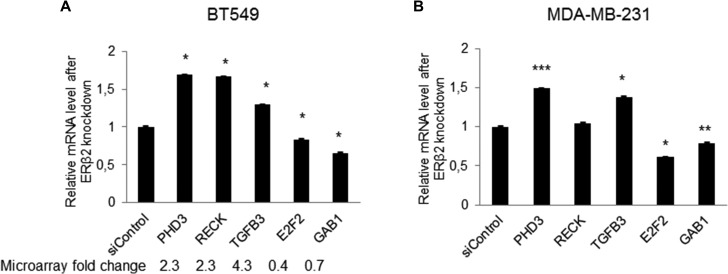
Validation of gene expression profiling data by qPCR (**A**) Real-time PCR analysis for a subset of ERβ2 regulated genes identified by microarray analysis in BT549 cells. mRNA levels are normalized to 36B4. Data represent means ± SD. *P < 0.05. Fold change derived from microarray analysis is presented as numbers below the bars. (**B**) Real-time PCR analysis of selected genes in MDA-MB-231 cells. mRNA levels are normalized to 36B4. Data represent means ± SD. *P < 0.05, **P < 0.01, ***P < 0.001. A, B, *p* values were calculated by *t*-test relative to control siRNA-treated cells.

qPCR analysis was performed to confirm changed expression of 5 genes (PHD3, RECK, TGFB3, E2F2, GAB1) identified as being regulated by ERβ2 in the global gene expression profiling analysis. These represent genes well known to be involved in cell growth, cell death, apoptosis, cell migration and invasion [21–26]. As shown in Figure 4A, the qPCR data confirmed the data from the microarray assay of BT549 cells. In addition, qPCR analysis of the expression of these genes was performed in the MDA-MB-231 cell line. Importantly, changed expression of 4 of the 5 genes was also observed in the MDA-MB-231 cell line upon ERβ2 knockdown, the exception being RECK which was not regulated in the MDA-MB-231 cell line upon ERβ2 knockdown (Figure 4B).

### ERβ2 promotes cell invasion potentially through repression of PHD3 with associated up-regulation of HIF-1α

**Figure 5:**
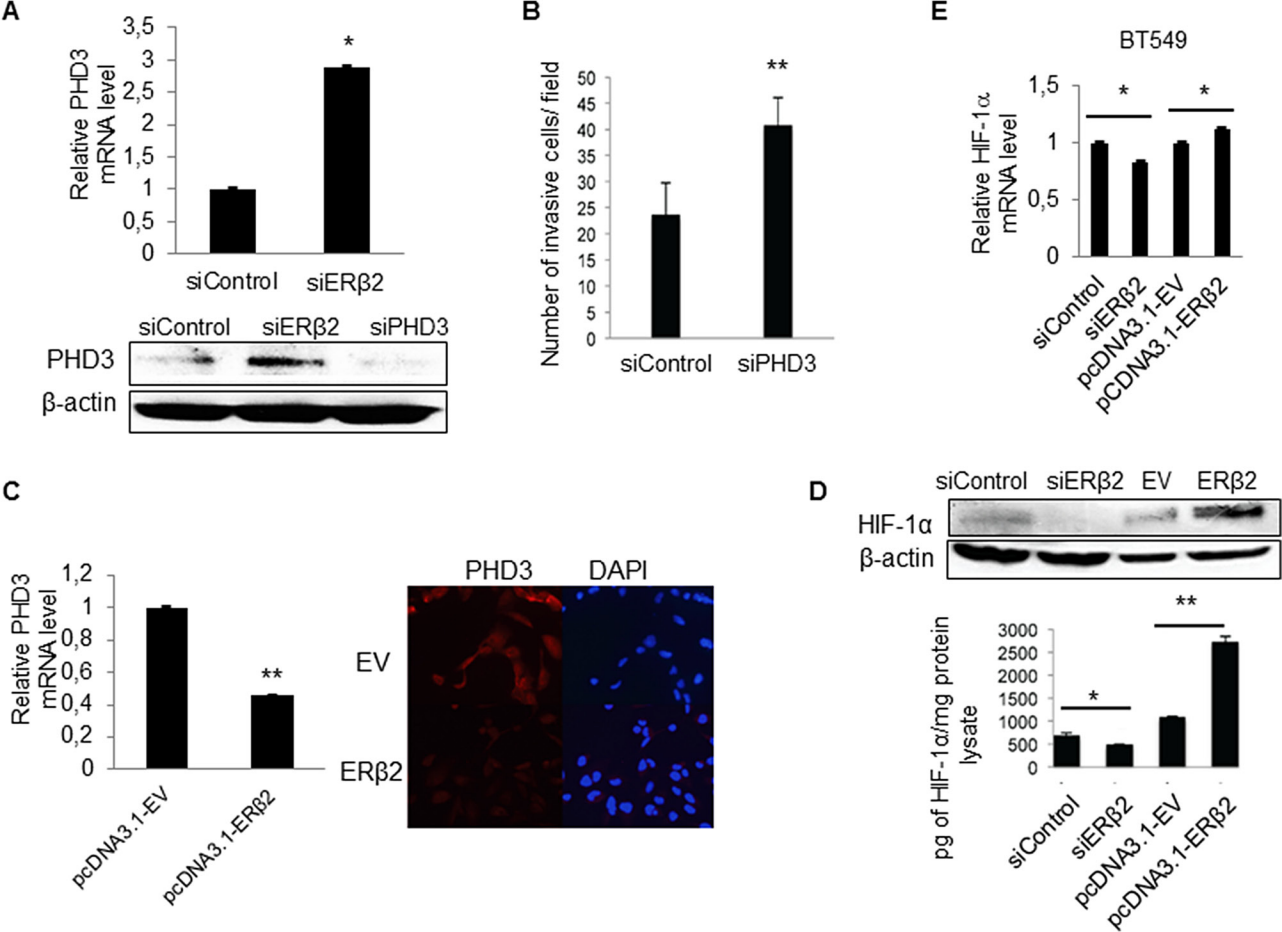
ERβ2 modulates levels of PHD3 and HIF-1α in TNBC cells (**A**) ERβ2 knock-down increases PHD3 mRNA and protein levels. BT549 cells were transfected with control siRNA or ERβ2 siRNA. RNA was collected after 48 h while protein was collected after 72 h. PHD3 mRNA levels were determined by a qPCR assay and was normalized to 36B4, *P < 0.05 (top panel) and PHD3 protein levels were determined by Western blot analysis. β-actin was used as a loading control (bottom panel). (**B**) PHD3 knockdown promotes cell invasiveness for the BT549 cell line. BT549 cells were transfected with control siRNA or PHD3 siRNA. Data represent means ± SD. **P < 0.01. (**C**) BT549 cells were transfected with ERβ2 containing plasmid or a control EV. After 24 h, RNA was collected; qPCR was used to determine the PHD3 mRNA level, that was normalized to 36B4 (bar chart). Data represent means ± SD. **P < 0.01. BT549 cells were seeded onto microscope cover slide after ERβ2 overexpression; 24 h after plating cells, PHD3 protein level was detected by immunofluorescence. DAPI was used for nuclear staining (right figure). (**D**) BT549 cells were transfected with control siRNA or ERβ2 siRNA, and with empty vector or ERβ2 containing plasmid. Protein lysates were collected and HIF-1α levels were evaluated by both Western blot analysis (top panel) and HIF-1α ELISA (bottom graph). *P < 0.05, **P < 0.01. *p* values were calculated by *t*-test. (**E**) qPCR analysis of HIF-1α mRNA level after overexpression or knockdown of ERβ2. BT549 cells were transfected with control siRNA or ERβ2 siRNA and with EV or ERβ2 containing plasmid. mRNA levels were normalized to 36B4. Data represent means ± SD. *P < 0.05. A,B,C,D,E, *p* values were calculated by *t*-test, relative to control siRNA or empty vector transfected cells.

PHD3, one of the most regulated genes upon ERβ2 knockdown is known to play a critical role in suppressing the growth of diverse tumor types. Figure 5A shows that PHD3 mRNA and protein levels are increased by ERβ2 knockdown in the BT549 cell line. Importantly, depletion of PHD3 results in increased cell invasiveness (Figure 5B). These findings suggest a link between ERβ2, PHD3 and cell invasion. Furthermore, in support of the link between ERβ2 and PHD3 expression, PHD3 mRNA and protein levels are reduced by overexpression of ERβ2 (Figure 5C). As PHD3 has been shown to promote degradation of HIF-1α [27] and HIF-1α has been shown to play key roles in many crucial aspects of breast cancer biology, including invasion and metastasis [28], we hypothesized that HIF-1α could be a mediator of the invasive effects of ERβ2 in this system. In support of this hypothesis, knockdown of ERβ2 decreased whereas overexpression of ERβ2 increased HIF-1α protein levels in BT549 cells as assayed by Western blot analysis and ELISA assays (Figure 5D). Knockdown and overexpression of ERβ2 also regulated the mRNA levels of HIF-1α with small fold changes (~1.2-fold) in BT549 cells (Figure 5E). In summary, our data suggest that ERβ2 decreased PHD3 gene expression and increased HIF-1α protein levels, thus contributing to the proliferative and invasive phenotype of TNBC cell lines.

### ERβ2 expression is high in breast tumor samples with low ERα expression and negatively correlates with PHD3 expression

**Figure 6:**
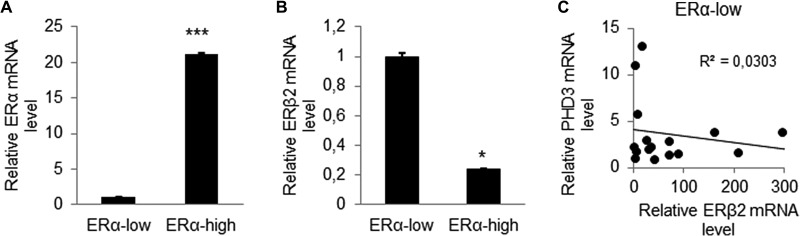
ERβ2 expression is high in breast tumor samples with low ERα expression and negatively correlates with PHD3 expression (**A**) qPCR analysis of ERα mRNA level of 50 human breast cancer tissues determined ERα-low expression (n=20) and ERα-high expression (*n* = 30) breast tumors. Data represent means ± SD. ***P < 0.001. (**B**) qPCR analysis of ERβ2 mRNA level in ERα-low expression tumor samples compared to ERα-high expression breast tumor samples. Data represent means ± SD. *P < 0.05. (**C**) Negative correlation of PHD3 mRNA expression to ERβ2 mRNA expression in ERα-low expression breast tumors. *P < 0.05.

To determine the clinical relevance of ERβ2 expression in breast cancer, we analysed ERβ2 expression in 50 human breast tumor samples. The tumor samples were divided into ERα-low expression (*n* = 20) and ERα-high expression (*n* = 30) based on ERα mRNA levels (*p* < 0.001) (Figure 6A). We observed that ERα-low expression tumors, that are generally more aggressive, express higher level of ERβ2 as compared to ERα-high expression tumors (Figure 6B) (*p* < 0.05). This is consistent with the high expression of ERβ2 that we report for TNBC cell lines. We further investigated the relationship between ERβ2 and PHD3 mRNA expression in the clinical samples. Our analysis showed a weak negative correlation between ERβ2 and PHD3 expression in ERα-low expression tumor samples (Figure 6C), which was not observed in ERα-high expression tumor samples (data not shown). These results support our findings in TNBC cell lines that knockdown of ERβ2 increases PHD3 expression. We did not find a correlation between ERβ2 and HIF-1α mRNA expression in clinical samples (data not shown). This may be due to the fact that the effect of ERβ2 on HIF-1α mRNA expression in cell lines is very modest (Figure 5E).

## DISCUSSION

The function of the human ERβ variant ERβ2 remains elusive. In this study we report that several TNBC cell lines express endogenous ERβ2 without expression of ERα and ERβ1. To our knowledge this is the first study that addresses the molecular mechanism and cellular phenotypes conferred by endogenous expression of ERβ2 alone, without the influence of ERα and ERβ1. We demonstrate that ERβ2 depletion significantly inhibited proliferation and invasion of TNBC cells. This was confirmed with a second set of siRNA (data not shown), supporting that this does not correspond to off-target effects. Moreover, we observed opposite cellular phenotypes and molecular changes resulting from overexpression of ERβ2, further supporting that the observed effects are related to ERβ2.

We used gene expression profiling to start to elucidate the molecular mechanisms associated with the proliferative and invasive properties of ERβ2 in the BT549 cell line and identified changes in pathways and molecular classes associated with cancer, cell cycle, cell survival and cell death compatible with a role of ERβ2 in cellular proliferation and invasion. To our knowledge, this study is the first to assay regulation of global gene expression by endogenous ERβ2.

In this study we propose that the proliferative and invasive phenotype associated with ERβ2 expression could be mediated via repression of PHD3 and subsequent up-regulation of HIF-1α. PHD3 is known to regulate HIF-1α by posttranslational modification. However, we also observe minor effects on HIF-1α mRNA levels upon modulation of ERβ2 levels. Indeed, studies also show that HIF-1α is additionally subject to transcriptional regulation [29]. It promotes cell proliferation, migration and invasion in various tumor cells [30–33]. PHD3 has been shown to exert anti-proliferative effects including inhibition of tumor growth in pancreatic cancer and renal cell carcinoma [34, 35]. High PHD3 expression is correlated with good clinical prognosis markers for breast cancer such as lower tumor grade, smaller tumor size and lower proliferation [5]. In a recent study, down-regulation of PHD3 expression was reported to occur during epithelial to mesenchymal transition (EMT) [36]. It remains unclear if ERβ2 regulates PHD3 directly or indirectly. In support of an indirect mechanism of ERβ2 regulation of PHD3, we were unable to detect binding of ERβ2 to the promoter region of the PHD3 gene using a ChIP assay (data not shown). Furthermore, our analysis revealed a weak negative correlation between ERβ2 and PHD3 mRNA expression in ERα-low expression breast tumors, consistent with the notion that ERβ2 downregulates PHD3. The weak correlation is presumably due to the small sample size and cell type heterogeneity of the tumor samples. Our data suggest that ERβ2 regulates PHD3 indirectly, which may also account for the poor correlation. Moreover, the lack of a correlation between ERβ2 and PHD3 expression in ERα-high expression tumor samples may be due to the low or even undetectable expression levels of ERβ2 in these tumor samples. Our data are consistent with Peurala et al. who found that PHD3 is mainly expressed in tumors with better prognosis and their preliminary data suggested that PHD3 negativity associates with triple negative tumors [6].

In this study we focus on ERβ2 regulation of PDH3. However, our analysis also identified additional ERβ2 target genes that could contribute to effects of ERβ2 on proliferation and invasion. Genes known to be involved in positive regulation of cell proliferation such as E2F2 [30] and GAB1 [29], were decreased upon knockdown of ERβ2 in both analysed TNBC cell lines. Furthermore, the regulators of cell cycle progression, cyclins E and A (CCNE2/CCNA2) were down regulated 1.3 and 1.4 fold, respectively, with a concomitant increase in the expression of p21WAF1/CIP1 (CDKN1A) of 1.5 fold (data not shown). Knockdown of ERβ2 also downregulated gene expression of the Wilms tumor-1 (WT-1) transcription factor known to play an important role in cellular development, cell survival and angiogenesis [32, 33]. Finally, genes involved in inhibition of cell migration and invasion such as RECK [26, 27] and TGFB3 [25] were induced by knockdown of ERβ2 in BT549 cells.

In this study, we screened the expression of ERβ2 in a panel of ten breast cancer cell lines. These cell lines can be classified into three different breast cancer subtypes according to the expression of ER, PR and HER2 receptor: ER-positive (MCF-7, MDA-MB-175, ZR-751 and CAMA-1), HER2-overexpressed (SK-BR-3 and HCC1569) and triple-negative subtypes (Hs578T, MDA-MB-231, BT549 and BT20). Gene expression profiling has further classified breast cancer into five subtypes: luminal A, luminal B, HER2-enriched, basal-like and normal-like [37]. The cell lines used in our study represent different breast cancer subtypes. For example, MCF-7 and ZR-751 cell lines are representative of luminal A and luminal B, respectively. SK-BR-3 closely resembles HER2-enriched, while the TNBC cell lines such as BT549, MDA-MB-231 and Hs578T resemble basal-like tumors. Clinically, the basal-like tumors are characterized by poorer survival outcomes and higher relapse rates than other subtypes of breast cancer [38]. Our results show that the highest level of endogenous ERβ2 expression was observed in the TNBC cell lines, indicating a role of ERβ2 in promotion of an aggressive phenotype. In line with this, our data also showed an increased expression of ERβ2 in ERα-low expression tumors compared with ERα-high expression tumors. TNBC lacks effective specific targeted therapy leading to poor survival. It is possible that a fraction of TNBC expresses ERβ2 and that this class of TNBC would benefit from therapeutic strategies targeting ERβ2. We were unable to detect endogenous ERβ2 protein in BT549 and MDA-MB-231 cells, that have the highest levels of ERβ2 mRNA expression, by Western blotting using the only specific ERβ antibody (PPZ0506) [17]. It is possible that the levels of ERβ2 are still not very high in these cell lines or that the antibody may not provide sufficient sensitivity.

Studies of ERβ2 are limited by the lack of this ERβ2 isoform in rodents. Thus it will be important to generate mouse models that express ERβ2 in different tissues. Furthermore, although ERβ2 does not bind tested ER ligands, it is possible that compounds that inhibit its function can be identified, thus providing tools to further explore the function of ERβ2 as well as be used therapeutic agents, for example in breast cancers that express ERβ2.

In summary, we identify TNBC breast cancer cell lines that express endogenous ERβ2 but not ERα or ERβ1 and show that ERβ2, when expressed alone in these cell lines, promotes cell proliferation and invasion and suggest a mechanism for these effects. Whether our results can be translated into an understanding of the molecular mechanism for the invasive nature of TNBC and potentially the identification of targeted therapies remains to be determined.

## MATERIALS AND METHODS

### Cell culture

Cell lines were obtained from the American Type Culture Collection (ATCC) and maintained at 37°C and 5% CO_2_. MCF-7, CAMA-1, SK-BR-3, Hs578T and BT20 cells were grown in DMEM medium (Gibco by Life Technologies, Carlsbad, CA) supplemented with 10% fetal bovine serum and 1% penicillin-streptomycin (Gibco); ZR-751, HCC1569, MDA-MB-231 and BT549 cells were grown in RPMI 1640 medium (Gibco) supplemented with 10% fetal bovine serum and 1% penicillin-streptomycin (Gibco); MDA-MB-175 cells were grown in L-15 medium (Gibco) supplemented with 10% fetal bovine serum and 1% penicillin-streptomycin (Gibco).

### RNA isolation and cDNA synthesis

Total RNA was extracted using the RNeasy Plus Kit (Qiagen, Hilden, Germany) according to the manufacturer's protocol. cDNA was synthesized using SuperScript™ VILO™ MasterMix according to the standard protocol (Invitrogen, Carlsbad, CA). In brief, 1 μg of RNA was mixed with 4 μl of SuperScript VILO MasterMix and DEPC treated water to a total volume of 20 μl. The reaction was gently mixed and incubated at 25°C for 10 mins, at 42°C for 60 mins and at 85°C for 5 mins. cDNA was stored at -20°C until further use.

### qPCR

Real-time PCR was performed with the SYBR Green I dye master mix (Applied Biosystems, Foster City, CA). qPCR reactions were analysed using a 7500 Fast Real-Time PCR System (Applied Biosystems) applying the following conditions: 95°C for 20 sec, followed by 40 cycles at 95°C for 3 sec and 60°C for 30 sec. Primer sequences for analysed genes were: ERβ1/ERβ2 forward 5′-TCCATGCGCCTGGCTAAC-3′, ERβ1 reverse 5′-CAGATGTTCCATGCCCTTGTTA-3′, ERβ2 reverse 5′-CCATCGTTGCTTCAGGCAA-3′, PHD3 forward 5′-GCCGGCTGGGCAAATACTA-3′, reverse 5′-CCGGATAGCAAGCCACCAT-3′, TGFB3 forward 5′-TACTATGCCAACTTCTGCTCAG-3′, reverse 5′-AACTTACCATCCCTTTCCTC-3′, GAB1 forward 5′-CCTGTTGCTCATCAACTGTCAAAGC-3′, reverse 5′-CTACACTCGATGTCCCAGATGGG-3′, E2F2 forward 5′-TGAGGACAAGGCCAACAAGAG-3′, reverse 5′-TTGCCAACAGCACGGATATC-3′, RECK forward 5′-AACAGGCCAACAGAACTTTTCAG-3′, reverse 5′-CATGTCATTCATGGCTCCTTGA-3′, ERα forward 5′-GCTACGAAGTGGGAATGATGAAAG-3′, reverse 5′-TCTGGCGCTTGTGTTTCAAC-3′. mRNA expression levels were normalized to acidic ribosomal phosphoprotein P0 (36B4) mRNA[39] reference gene forward 5′-GTGTTCGACAATGGCAGCAT-3′, reverse 5′-GACACCCTCCAGGAAGCGA-3′ or to glyceraldehyde 3-phosphate dehydrogenase (GAPDH) reference gene forward 5′-GACCCCTTCATTGACCTCAACT-3′, reverse 5′-GAATTTGCCATGGGTGGAAT-3′.

### Plasmids and siRNA transfection

The ERβ2 cDNA was cloned into the pcDNA3.1 vector with a FLAG tag at the N-terminus. MDA-MB-231 or BT549 cells at a confluency of about 70-90 % were transfected with 2.5 μg of either pcDNA3.1-ERβ2 or pcDNA3.1-empty vector (pcDNA3.1-EV) plasmid using 5 μl/well of Lipofectamine^®^2000 according to manufacturer's instructions (Invitrogen, Carlsbad, CA). For siRNA transfection, BT549 and MDA-MB-231 cells were transfected at about 30-50 % confluency with two different sets of siRNA targeting ERβ2 mRNA (Thermo Scientific and Dharmacon) or control siRNA (Sigma) and siRNA targeting PHD3 mRNA with the sequence GUACUUUGAUGCUGAAGAAUU (Sigma) using 10 μl/well of INTERFERin^TM^ siRNA transfection reagent (Polyplus, Illkirch, France) according to manufacturer's instructions.

### Western blot and ELISA assay

Protein lysates were extracted using RIPA buffer (Thermo Fisher, Waltham, MA) with protease inhibitor cocktail (Roche, Basel, Switzerland). Thirty μg of protein was boiled with 5x Laemli buffer (0.5 M Tris-HCl, 10% SDS, 30% glycerol, 0.25% Bromphenol blue, 20% β-mercaptoethanol) for 10 mins. Protein samples were separated by SDS-PAGE, and electrophoretically transferred to nitrocellulose membranes (GE Healthcare, Danderyd, Sweden). After milk blocking, blots were incubated over night at 4°C with the following primary antibodies: PPZ0506 [17] for ERβ2 (R&D systems, Abingdon, UK), PHD3 (Novus biological, Littleton, CO), HIF-1α (Pharmingen, San Diego, CA) and β-actin (Sigma Aldrich, Dorset, UK). Secondary antibodies were anti-mouse and anti-rabbit IgG (GE Healthcare, Danderyd, Sweden).

For ELISA analysis, thirty μg of protein was used. HIF-1α ELISA was performed using Human/Mouse Total HIF-1 alpha DuoSet IC ELISA kit (R&D systems, Abingdon, UK) according to manufacturer's instructions.

### Immunofluorescence

100000 cells were seeded on microscope cover glass (Menzel Glaser, Germany) 24 h after ERβ2 or control plasmid transfection. The next day, immunofluorescence analysis was performed as described previously [40]. PHD3 (Novus biological, Littleton, CO) antibody was used. DAPI (4′, 6-diamidino-2-phenylindole) (Sigma Aldrich, Dorset, UK) was used at 0.5 μg/mL for nuclear staining. The coverslips were then mounted on a microscope slide SuperFrost^®^Plus (Menzel Glaser, Germany) with fluorescence mounting solution (Dako, Carpinteria, CA).

### Cell proliferation assay

3000 cells per well were seeded in 96-well plates 24 h after ERβ2 or control plasmid transfection and 48 h after ERβ2 siRNA or control siRNA transfection. Cell proliferation was determined after 1, 2, 3 or 4 days using the WST-1 kit (Roche, Basel, Switzerland) according to the instructions of the manufacturer. The absorbance was measured at 450 nm using NanoQuant spectrophotometer (Tecan, Männedorf, Switzerland).

### Invasion assay

Twenty-four hours after transfection of control siRNA or ERβ2 siRNA and pcDNA3.1-empty vector or pcDNA3.1-ERβ2, approximately 2.5 × 10^4^ cells were seeded in the upper chambers of 24-well BD Biocoat growth factor-reduced Matrigel Invasion Chambers (Becton Dickinson, Bedford, MA) with media without FBS and incubated at 37°C for 24 h. Medium containing 10% FBS was added to the lower chamber. Non-invading cells in the upper chamber were removed by scraping with cotton sticks and washed with PBS. Cells that migrated to the lower chamber were fixed and stained using Gurr for microscopy (VWR, Radnor, PA) and counted under a microscope Zeiss Axiovert S100 under magnification ×20.

### Gene expression microarray analysis

Total RNA from three biological replicates were hybridized to Affymetrix Human Gene 1.1 ST arrays, which contain probes for 33299 gene sequences. Experimental steps such as probe synthesis, hybridization and scanning were done according to the Affymetrix protocol (www.affymetrix.com). Pre-processing for background correction/normalization was performed in the Affymetrix Expression Console using the Robust Multichip Average (RMA) method [41]. Two-tailed Student's *t*-test was used to derive *p*-values, and the false discovery rates were estimated using the q-value. A cut-off fold change of at least 1.5 and *p* value < 0.05 were used to define differentially regulated genes. The q-value, representing the false discovery rate, was less or equal to 0.1. The microarray raw data are deposited in GEO (accession number GSE57379).

To derive genes as input for pathway analysis the following cut-offs were applied; fold change of ≥ 2, *p* value < 0.05 and q value ≤ 0.1. The IPA software (www.ingenuity.com) was used for this analysis. The entire IPA core analysis was performed based on information in the Ingenuity Pathway Knowledge Base (IPKB), which derives information from known relationships of molecules, functions and interactions of genes published in the literature.

IPA analysis identified pathways that were most significantly changed upon ERβ2 knock down. Associated network functions and molecular functions are two of the categories used for enrichment testing within IPA. Biological functions were ranked according to the significance of that function to the network. Fisher's exact test was used to derive the *p*-values in IPA.

### Human breast tumor samples

The tumor samples in this study have been previously described [18]. The studies were approved by the ethical committee of the Karolinska Institute.

### Statistics

Student's *t*-test was used to determine statistically significant differences and *p* < 0.05 was considered to be significant unless otherwise specified.

## SUPPLEMENTARY MATERIALS FIGURES AND TABLES



## Abbreviations

ER: estrogen receptor
ERβ2: estrogen receptor beta isoform 2
ERβ1: estrogen receptor beta isoform 1
ERα: estrogen receptor alpha
PHD3: prolyl hydroxylase 3
HIF-1α: hypoxia inducible factor 1α
TGFB3: transforming growth factor beta 3
E2F2: E2F transcription factor 2
GAB1: GRB2-associated-binding protein 1
RECK: Reversion-inducing cysteine-rich protein with Kazal motifs
AF-2: activation function 2
RMA: robust multichip average
IPKB: ingenuity pathway knowledge base
TNBC: triple-negative breast cancer
EMT: epithelial-to-mesenchymal transition
PR: progesterone receptor
HER2: human epidermal growth factor receptor 2
EV: empty vector
