# Supplementary material for: Estrogen receptor β2 induces proliferation and invasiveness of triple negative breast cancer cells: association with regulation of PHD3 and HIF-1α
Source: Oncotarget. 2017 Sep 4;8(44):76622–33. doi: 10.18632/oncotarget.20635 (PMC5652730; doi:10.18632/oncotarget.20635)
Supplement: Supplementary file 1 [file oncotarget-08-76622-s001.pdf]

Estrogen receptor β2 induces proliferation and invasiveness of triple negative breast cancer cells: association with regulation of PHD3 and HIF-1α

SUPPLEMENTARY MATERIALS

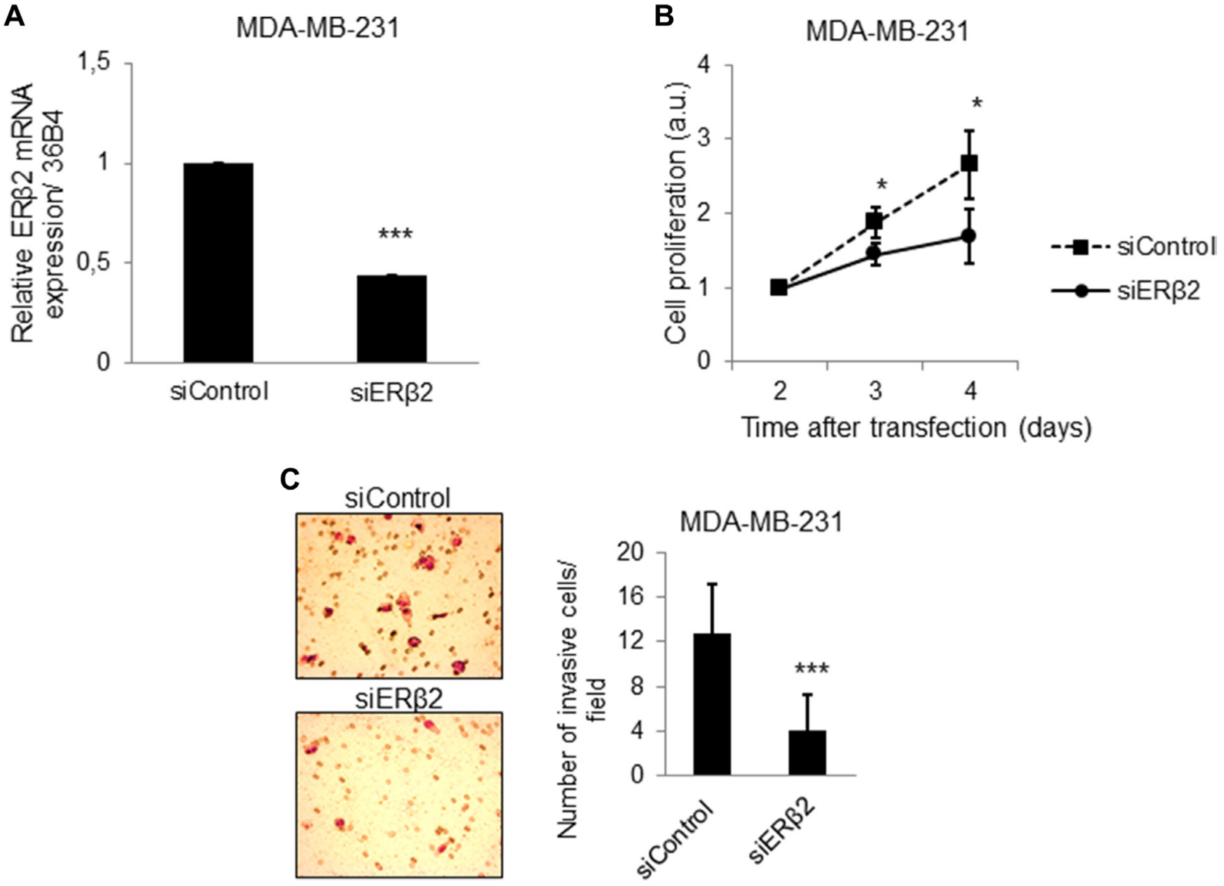

Supplementary Figure 1: (**A**) ERβ2 siRNA down-regulates ERβ2 mRNA in MDA-MB-231 cells. ERβ2 mRNA level was determined by qPCR after transfection with control siRNA or ERβ2 siRNA. Data are normalized to 36B4 and shown as relative fold change compared to control siRNA ± SD. ***$P < 0.001$. (**B**) ERβ2 depletion reduces proliferation of the MDA-MB-231 cell line. MDA-MB-231 cells were transfected with control siRNA or ERβ2 siRNA. WST-1 assays as a measure of cellular proliferation were carried out at the indicated time points after siRNA transfection. Data are shown as means ± SD. *$P < 0.05$. The experiment was repeated three times. One representative experiment is shown. (**C**) ERβ2 depletion reduces invasion of MDA-MB-231 cell line. MDA-MB-231 cells were transfected with control siRNA or ERβ2 siRNA, and cell invasion was evaluated by the BD Biocoat growth factor reduced Matrigel invasion chamber assay. Data represent means ± SD. ***$P < 0.001$. Experiment was repeated twice. One representative experiment is shown. A,B,C, *p* values were calculated by *t*-test.

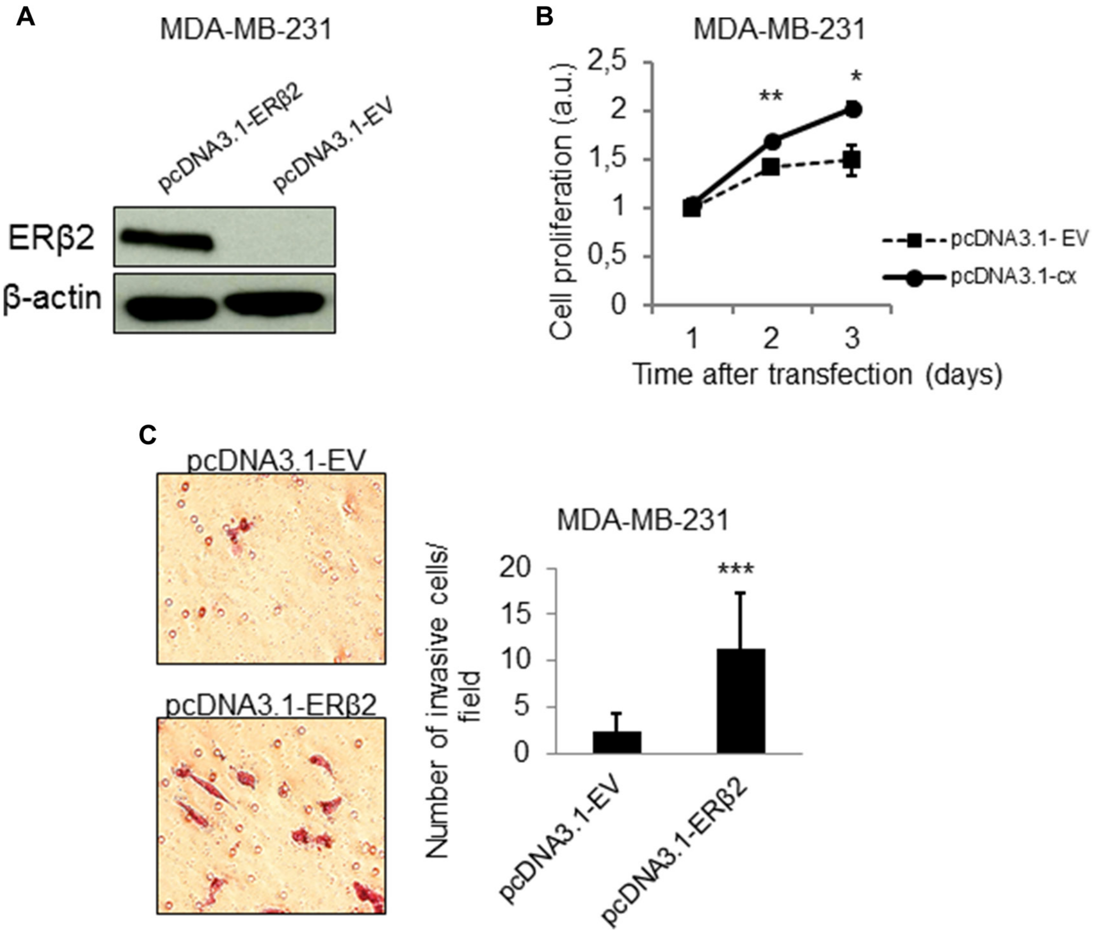

Supplementary Figure 2: (**A**) Western blot analysis showing increased protein level of ERβ2 after transient overexpression of ERβ2 protein. ERβ2 was detected by the PPZ0506 antibody. β-actin was used as a loading control. (**B**) ERβ2 overexpression promotes cell proliferation in MDA-MB-231 cells. WST-1 assays of cell proliferation were carried out at the indicated time points after transfection of ERβ2 or empty vector (EV). Ratio of absorbance to day 1 is calculated. Data are shown as means of relative absorbance ± SD. $^*P < 0.05$, $^{**}P < 0.01$. Experiments were repeated tree times. One representative experiment is shown. (**C**) ERβ2 overexpression promotes cell invasion in the MDA-MB-231 cell line. MDA-MB-231 cells were transfected with ERβ2 or EV, and cell invasion was evaluated by the BD Biocoat growth factor reduced Matrigel invasion chamber assay. Data represent means ± SD. $^{***}P < 0.001$. Experiment was repeated twice. One representative experiment is shown. B,C, *p* values were calculated by *t*-test.
